
JOURNAL INFORMATION
==============================
NLM Title Abbreviation: ScientificWorldJournal
Journal ID: ScientificWorldJournal
NLM Journal ID: 101131163
Journal ID: TSWJ

The Scientific World Journal

ISSN: 2356-6140
EISSN: 1537-744X
Publisher: Wiley

ARTICLE INFORMATION
==============================
PMCID: PMC6009382
PMID: 29973818
DOI: 10.1100/tsw.2002.44
Article ID: 529750
Article version: 1
Subjects: Short Report

Approaches to Analyze Protein Interactions

Fields Stanley 1 2 *
Drees Becky 1
Hazbun Tony R. 1 2
Tucker Chandra 1
Tong Amy Hin Yan 3
Evangelista Marie 3
Boone Charles 3
Nardelli Giuliano 4
Castagnoli Luisa 4
Zucconi Adriana 4
Cesareni Gianni 4
Bader Gary 5
Hogue Chris 5
Departments of Genome Sciences and Medicine, University of Washington, Box 357730, Seattle, WA 98195, USA
Howard Hughes Medical Institute, University of Washington, Box 357730, Seattle, WA 98195, USA
Banting and Best Department of Medical Research, University of Toronto, Toronto, Ontario, Canada M5G 1L6,
Department of Biology, University of Rome Tor Vergata, 00133 Rome, Italy
Samuel Lunenfeld Research Institute, 600 University Avenue, Toronto, Ontario, Canada M5G 1X5,
*Stanley Fields: fields@u.washington.edu
Publication date: 2002
Electronic publication date: 2002 Jan 5
Volume: 2
First page: 93
Last page: 94
Copyright: Copyright © 2002 Stanley Fields et al.
Copyright year: 2002
License: This is an open access article distributed under the Creative Commons Attribution License, which permits unrestricted use, distribution, and reproduction in any medium, provided the original work is properly cited.
License URL: https://creativecommons.org/licenses/by/3.0/

Funding: Howard Hughes Medical Institute

==============================
Advances in Gene Technology: The Genome and Beyond –
Structural Biology for Medicine (Proceedings of the 2002 Miami
*Nature Biotechnology* Winter Symposium)
*TheScientificWorld* 2002, 2(S2), 93–94
ISSN 1532-2246; DOI 10.1100/tsw.2002.44

### APPROACHES TO ANALYZE PROTEIN INTERACTIONS

Stanley Fields[1,2,]*, Becky Drees[1], Tony R. Hazbun[1,2], Chandra Tucker[1], Amy Hin Yan Tong[3], Marie Evangelista[3], Charles Boone[3], Giuliano Nardelli[4], Luisa Castagnoli[4], Adriana Zucconi[4], Gianni Cesareni[4], Gary Bader[5], and Chris Hogue[5]
[1]Departments of Genome Sciences and Medicine, [2]Howard Hughes Medical Institute, University of Washington, Box 357730, Seattle, WA 98195; [3]Banting and Best Department of Medical Research, University of Toronto, Toronto, Ontario, Canada M5G 1L6; [4]Department of Biology, University of Rome Tor Vergata, 00133 Rome, Italy;. [5]Samuel Lunenfeld Research Institute, 600 University Avenue, Toronto, Ontario, Canada, M5G 1X5
*fields@u.washington.edu

**INTRODUCTION.** Proteins often work as parts of large macromolecular assemblies that carry out cellular processes. Understanding protein function is advanced by the identification of the interactions of proteins with other proteins or with other ligands such as nucleic acids or small molecules. We have been interested in designing and implementing simple yeast-based assays to identify or characterize these interactions.

**METHODS.** A yeast activation domain array was screened as in Uetz et al.[1]. Phage display and computational prediction of protein networks for yeast SH3 domains is described in Tong et al.[2]. Gel shift assays were carried out using a fragment of the *SUC2* promoter and yeast extracts of pooled glutathione S-transferase (GST) fusions[3]. The yeast biosensor and its use in identifying mutations or ligands are described in Tucker and Fields[4].

**RESULTS AND DISCUSSION.** We have generated an array of ~6000 yeast transformants, each designed to express one of the open reading frames (ORFs) of *Saccharomyces cerevisiae* as a Gal4 activation domain fusion for use in the two-hybrid assay[1]. This array has been screened against >700 proteins to identify over a thousand potential protein-protein interactions. In one approach, we determined the protein interaction partners for a peptide recognition module[2]; genome sequences have identified numerous members of these module families.

Our approach consists of obtaining two sets of predicted networks: one from the computational prediction of interacting proteins based on peptide ligand data from phage display and another from the experimental identification of interactions from large-scale two-hybrid interactions. Using this strategy with SH3 domains (which recognize proline-containing peptides) from yeast, computational analysis of the hypothetical and experimental networks showed a substantial intersection of the data sets. This analysis identified a set of 53 highly likely SH3-domain interactions, several of which have been confirmed *in vivo* by coimmuno-precipitation experiments.

To identify DNA-protein interactions on a genomewide level, we have applied a biochemical genomics strategy[3]. An array of ~6000 yeast strains, each containing a different yeast ORF fused to GST, is grown in defined pools and GST-ORFs are purified; pools are assayed for activities, and active pools are deconvoluted to identify the source strains. We used this approach with a DNA fragment from the yeast *SUC2* promoter and characterized ten yeast proteins with binding activity for this fragment, of which three appear to be specific.

We have developed a biosensor that reports the binding of small-molecule ligands to proteins as changes in growth of temperature-sensitive yeast. The yeast lack dihydrofolate reductase (DHFR) and are complemented by mouse DHFR containing a ligand-binding domain inserted in a flexible loop. With either the FKBP12 protein (which binds FK506) or the estrogen receptor $\alpha$ (ER$\alpha$), yeast show increased growth in the presence of the corresponding ligand. We used this sensor to identify mutations implicated in ligand binding, and to screen a chemical array to identify ligands that bind to FKBP12 or ER$\alpha$.

**ACKNOWLEDGMENT.** This work was supported by grants to C.B., G.C., and S.F. S.F. is an investigator of the Howard Hughes Medical Institute.

**REFERENCES**

1. Uetz, P., Giot, L., Cagney, G., Mansfield, T.A., Judson, R.S., Narayan, V., Lockshon, D., Srinivasan, M., Pochart, P., Qureshi-Emili, A., Li, Y., Godwin, B., Conover, D., Kalbfleisch, T., Vijayadamodar, G., Yang, M., Johnston, M., Fields, S., and Rothberg, J.M. (2000) Nature 403, 623–627.
2. Martzen, M.R., McCraith, S.M., Spinelli, S.L., Torres, F.M., Fields, S., Grayhack, E.J., and Phizicky, E.M. (1999) Science 286, 1153–1155.
3. Tong, A.H.Y., Drees, B., Nardelli, G., Bader, G.D., Castagnoli, L., Evangelista, M., Ferracuti, S., Nelson, B., Paoluzi, S., Quondam, M., Zucconi, A., Hogue, C., Fields, S., Boone, C., and Cesareni, G. (2001) Science, in press.
